# Machine Learning Analysis of RNA-Seq Data Identifies Key Gene Signatures and Pathways in Mpox Virus-Induced Gastrointestinal Complications Using Colon Organoid Models

**DOI:** 10.3390/ijms252011142

**Published:** 2024-10-17

**Authors:** Mostafa Rezapour, Aarthi Narayanan, Metin Nafi Gurcan

**Affiliations:** 1 Center for Artificial Intelligence Research, Wake Forest University School of Medicine, Winston-Salem, NC 27101, USA; mgurcan@wakehealth.edu; 2 Department of Biology, George Mason University, Fairfax, VA 22030, USA; anaraya1@gmu.edu

**Keywords:** Mpox virus (MPXV), gastrointestinal complications, colon organoids, machine learning, RNA-seq analysis, differential gene expression

## Abstract

Mpox, caused by the Mpox virus (MPXV), emerged globally in 2022 with the Clade IIb strain, presenting a critical public health challenge. While MPXV is primarily characterized by fever and rash, gastrointestinal (GI) complications, such as diarrhea and proctitis, have also been observed. This study is a reanalysis of GSE219036 without own data and focuses on the impact of MPXV infection on the colon, using human-induced pluripotent stem cell-derived colon organoids as a model. We applied a tailored statistical framework for RNA-seq data, Generalized Linear Models with Quasi-Likelihood F-tests and Relaxed Magnitude–Altitude Scoring (GLMQL-RMAS), to identify differentially expressed genes (DEGs) across MPXV clades: MPXV I (Zr-599 Congo Basin), MPXV IIa (Liberia), and MPXV IIb (2022 MPXV). Through a novel methodology called Cross-RMAS, we ranked genes by integrating statistical significance and biological relevance across all clades. Machine learning analysis using the genes identified by Cross-RMAS, demonstrated 100% accuracy in differentiating between the different MPXV strains and mock samples. Furthermore, our findings reveal that MPXV Clade I induces the most extensive alterations in gene expression, with significant upregulation of stress response genes, such as *HSPA6* and *FOS*, and downregulation of genes involved in cytoskeletal organization and vesicular trafficking, such as *PSAP* and *CFL1*. In contrast, Clade IIb shows the least impact on gene expression. Through Gene Ontology (GO) analysis, we identified pathways involved in protein folding, immune response, and epithelial integrity that are disrupted in infected cells, suggesting mechanisms by which MPXV may contribute to GI symptoms.

## 1. Introduction

Mpox, formerly known as Monkeypox, is a zoonotic infectious disease caused by the Mpox virus (MPXV), a double-stranded DNA virus belonging to the Orthopoxvirus genus within the Poxviridae family [1]. MPXV shares its genus with several significant viruses, including the variola virus, known for causing smallpox [2]. The disease was first identified in humans in 1970 in the Democratic Republic of the Congo and has since been detected in various other regions, traditionally confined to parts of central and west Africa [1]. Over the years, Mpox has evolved into two primary clades, I and II (IIa and IIb): the Congo Basin (Clade I), West Africa (Clade IIa), and the recently identified 2022 MPXV (Clade IIb) [3].

The recent global escalation of Mpox began with an outbreak of the Clade IIb strain in 2022, marking a significant spread across over 120 countries as of August 2024 [1]. This widespread transmission has resulted in over 100,000 laboratory-confirmed cases and more than 220 fatalities, prompting the World Health Organization to declare Mpox a public health emergency of international concern twice, the latest in August 2024 [1]. The ongoing research and public health response aim to reduce the transmission through enhanced surveillance, vaccination strategies, and community engagement amidst challenges of stigma and discrimination that threaten to undermine control efforts [1].

MPXV primarily spreads through close, personal contact, including direct skin-to-skin contact with an infected person’s rash, scabs, or bodily fluids, and through saliva or respiratory secretions [4]. It can also be transmitted during intimate activities, like oral, anal, or vaginal sex, as well as hugging, kissing, or touching objects contaminated by an infected person, such as clothing, bedding, and towels [4]. Pregnant individuals can pass the virus to their fetus, and while animal-to-human transmission can occur through contact with infected animals or their fluids, human-to-human respiratory transmission is considered low [4].

While the most reported symptoms of Mpox infection are fever, headache, muscle aches, back pain, and a distinct pustular rash [1], there are also significant, albeit less frequent, complications, such as secondary bacterial infections [5], oral ulcers [6], and gastrointestinal (GI) issues [7]. The GI symptoms observed in patients include nausea, diarrhea characterized by increased stool water content, abdominal pain situated between the chest and the groin, and various rectal complications [8]. These rectal issues include bleeding, severe pain, rectal perforation (also known as bowel perforation), painful defecation, and proctitis, which encompasses inflammation of the rectum and anus, potentially extending to include rectitis in clinical discussions [9]. Additionally, patients may experience tenesmus, which is a distressing, continual urge to defecate, even with an empty colon [7].

Such GI manifestations are crucial to understand due to their impact on patient management and potential long-term outcomes if not properly treated. Unaddressed, these symptoms can escalate to severe complications including sepsis, dehydration, encephalitis, blindness, acute respiratory distress syndrome, and even hemorrhagic disease [7]. Ramakrishnan et al. [10] conducted a systematic review that examined these GI manifestations associated with Mpox. Following the Preferred Reporting Items for Systematic Reviews and Meta-Analyses (PRISMA) guidelines, their review included 33 papers encompassing 830 patients, revealing that significant GI symptoms such as proctitis, vomiting, diarrhea, rectal pain, nausea, tenesmus, rectal bleeding, and abdominal pain are common among Mpox patients. This comprehensive examination emphasizes the importance for healthcare professionals to recognize these GI manifestations to improve understanding and management of Mpox pathophysiology.

Our study aims to explore further the impacts of MPXV on the colon, contributing to severe GI and systemic complications observed in infected patients. Historical research on MPXV has predominantly employed various cell lines and animal models, such as BSC-40, LLC-MK2, and Vero cells, to conduct in vitro infection experiments and pharmacological developments [11,12]. However, these models, derived from monkey cells, do not precisely mimic human pathophysiological responses to MPXV infection. In a significant advancement, Watanabe et al. [13] introduced the use of pluripotent stem (iPS) cell-derived colon organoids as models. These organoids represent the primary presumed sites of MPXV infection, providing a unique platform to examine organ-specific tropism, host immune responses, and tissue-specific pathology across MPXV Clades I, IIa, and the 2022 (IIb) variant.

Despite the valuable RNA-seq data extracted from colon organoids infected with different MPXV strains and generated by Watanabe et al. [13], there remains an opportunity to further leverage the capabilities of RNA sequencing data by employing more proper statistical and machine learning methods. Watanabe et al. [13] primarily focused on viral growth efficiency and host responses in both human keratinocytes and colon organoids, yet their analysis of the colon organoid data was less comprehensive. They concentrated more on comparing infection efficiency and cellular responses between keratinocytes and organoids rather than conducting a detailed investigation of specific gene expression changes within the colon organoids. Additionally, their study used Student’s *t*-test [14] and ANOVA [15] that may not fully capture the complexities inherent in RNA-seq datasets because these statistical tests assume a normal distribution of data and equal variance among groups, conditions that are rarely met in RNA-seq datasets.

RNA-seq data are typically characterized by non-normal distribution and variance that is dependent on the mean expression level, leading to potential biases and incorrect interpretations of gene expression differences when using these tests, e.g., a *t*-test. In the realm of RNA-seq analysis, it is widely acknowledged that tests assuming normality can lead to substantial errors in type I and type II error rates [16]. Marioni et al. [17] and Robinson and Oshlack [18] have demonstrated that methods taking into account the discrete nature and distribution of RNA-seq data, such as those based on negative binomial distributions, provide more reliable results.

However, building on the valuable RNA-seq data generated by Watanabe et al. [13], our study employs an enhanced statistical and machine learning analytical framework designed to overcome the limitations of traditional statistical methodologies for RNA-seq data analysis. By employing Generalized Linear Models with Quasi-Likelihood F-tests and Relaxed Magnitude–Altitude Scoring (GLMQL-RMAS), whose different variants have been applied and validated in our previous studies [19,20,21,22], we provide a more robust approach to the analysis of gene expression data.

GLMQL-RMAS has demonstrated its superiority over traditional gene ranking methods in several independent studies across a range of biological contexts. These studies consistently show that GLMQL-RMAS not only improves the accuracy of gene selection but also provides a robust framework for addressing complex biological questions.

In our recent study [19] on human lung organoids in response to influenza A virus (IAV), human metapneumovirus (MPV), and parainfluenza virus type 3 (PIV3) infections, we demonstrated that GLMQL-RMAS is superior to traditional methods employed in EdgeR or DESeq2 for ranking genes, which typically use *p*-values or LogFC. Our findings revealed that GLMQL-RMAS identified only three genes capable of differentiating all mock and infected samples at two post-infection time points: 24 and 72 h (see Figure 7 in [19]). In contrast, when using EdgeR and DESeq2 based on either *p*-value ranking (see Figure 8 in [19]) or LogFC ranking (see Figure 9 in [19]), GLMQL-RMAS proved to be more effective than these traditional methods for ranking.

In another study [20] aimed at addressing the methodological challenges commonly encountered in RNA-seq data analysis within cancer studies, GLMQL-RMAS effectively identified genes capable of differentiating between subjects with positive axillary lymph node metastasis and those without (see Figure 5 in [20]). These genes were subsequently validated through GO and GSEA hallmark pathway analyses. The ranking system of GLMQL-RMAS was also tested and validated in another study involving Ebola-infected nonhuman primates [22]. The top selected gene by our method was capable of differentiating positive from negative samples in a held-test set with 100% accuracy, while the best performance by EdgeR or DESeq2 was 72% using either *p*-value or LogFC (see Table 2 in [22]). This study differs from Watanabe et al. [13] in the following ways:

1. While Watanabe et al. [13] relied on statistical methods, such as Student’s *t*-test [14] or ANOVA [15] for RNA-seq data, we employed GLMQL-RMAS [19,20,21,22]. Our application of GLMQL-RMAS directly addresses the inherent non-normal distribution and overdispersion found in RNA-seq data, which traditional methods, like Student’s *t*-test [14] or ANOVA [15], fail to manage effectively. GLMQL-RMAS employs Generalized Linear Models (GLMs) [23] and a quasi-likelihood estimation [24] that adapts to unique characteristics of RNA-seq data, thus providing a more accurate reflection of the true biological variations across samples. Relaxed Magnitude–Altitude Scoring (RMAS) enhances this by prioritizing genes not just based on statistical significance but also considering the biological magnitude of their changes, ensuring that the identified biomarkers are both statistically significant and biologically relevant.
2. While Watanabe et al. [13] determined gene expression levels using transcripts per kilobase million (TPM) [25], we are employing Trimmed Mean of M-values (TMM) normalization [18]. TMM has been shown to be more effective than TPM for differential expression (DE) analysis in RNA-seq data, particularly in addressing RNA composition biases across samples [18,26,27,28]. Robinson and Oshlack [18] demonstrated that TMM normalization results in lower false discovery rates and improved accuracy in detecting differentially expressed genes. Zhao et al. [26] showed that TPM is not suitable for cross-sample comparisons and differential expression (DE) analysis due to issues with transcript distribution differences and sequencing depth. Abbas-Aghababazadeh et al. [27] highlighted that TMM normalization is effective in handling RNA composition differences, which TPM may fail to address. Zhao et al. [28] highlighted that TPM normalization was often misused for cross-sample comparisons due to differences in RNA composition and sequencing protocols, leading to misleading results. They emphasized that TPM reflected relative abundance within a sample and should not have been used for differential expression analysis when total RNA content and distributions differed across samples.
3. The third difference between our study and Watanabe et al. [13] lies in how we approach Gene Ontology (GO) analysis. While Watanabe et al. [13] used DAVID-based GO enrichment analysis for genes specifically altered by the 2022 MPXV strain in comparison to the other strains, our approach focuses on a more granular analysis using the clusterProfiler 4.0 package [29] in R. Unlike Watanabe et al. [13], who used strain comparisons, we employed mock samples as a baseline to identify significant genes for each clade for GO analysis.
4. The fourth difference in our study is the introduction of a novel methodology, namely Cross-RMAS. This method ranks genes across three statistical contrasts simultaneously, comparing all MPXV clades against mock samples. Cross-RMAS is designed to identify unique and common genes across all possible combinations of contrasts and rank them by prioritizing those with the maximum log fold change (LogFC) and the smallest *p*-value across all strains where the gene is identified as significant. By categorizing genes into seven distinct groups, ranging from unique to a single clade to common across all clades, this method enables a comprehensive analysis of gene expression. It effectively prioritizes genes that demonstrate consistent statistical significance and biological relevance across the comparisons, providing a powerful tool for identifying top biomarkers.
5. The final difference in our study is the application of machine learning models to distinguish between various strains of MPXV and mock samples using biomarkers identified through the GLMQL-RMAS approach. In this analysis, we employ key upregulated genes, selected via the Cross-RMAS method, as input features for supervised models, such as Logistic Regression (LR) [30,31] and Support Vector Machine (SVM) [32], with a linear kernel. These models are specifically chosen for their effectiveness with small datasets, like our study’s three samples per condition. To enhance model reliability and minimize overfitting, we apply k-fold stratified cross-validation (k = 3) or leave-one-out cross-validation.

## 2. Results

### 2.1. GLMQL-RMAS: Generalized Linear Models with Quasi-Likelihood F-Tests and Relaxed Magnitude–Altitude Scoring

**Figure 1:**
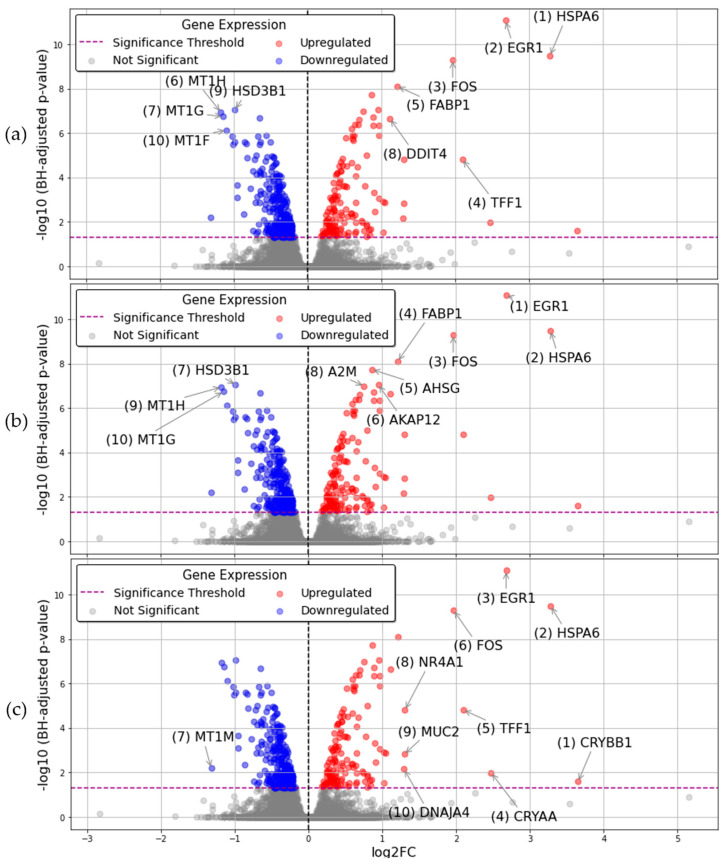
This figure presents volcano plots to demonstrate the efficacy of different gene ranking systems following GLMQL application on MPXV I-infected samples versus mock samples: (**a**) MAS ranking, which integrates both log fold change (LogFC) and BH-adjusted *p*-values to prioritize genes; (**b**) ranking based solely on BH-adjusted *p*-values, highlighting genes with the most statistical significance irrespective of effect size; and (**c**) ranking based solely on LogFC, emphasizing genes with the greatest expression changes without considering statistical significance.

The GLMQL-RMAS methodology consists of two components: Generalized Linear Models with Quasi-Likelihood F-Tests (GLMQL), ideally suited for multiple hypothesis testing in RNA-seq data, and Relaxed Magnitude-Altitude Scoring (RMAS), which ranks significant genes by maximizing log fold change (LogFC) and minimizing *p*-values simultaneously. To illustrate the efficacy of RMAS in gene selection, Figure 1 displays volcano plots based on three different rankings after applying GLMQL to compare MPXV I-infected samples against mock samples (baseline). (a) MAS integrates both LogFC and the BH-adjusted *p*-value, (b) is solely based on the BH-adjusted *p*-value, and (c) is solely based on LogFC.

**Figure 2:**
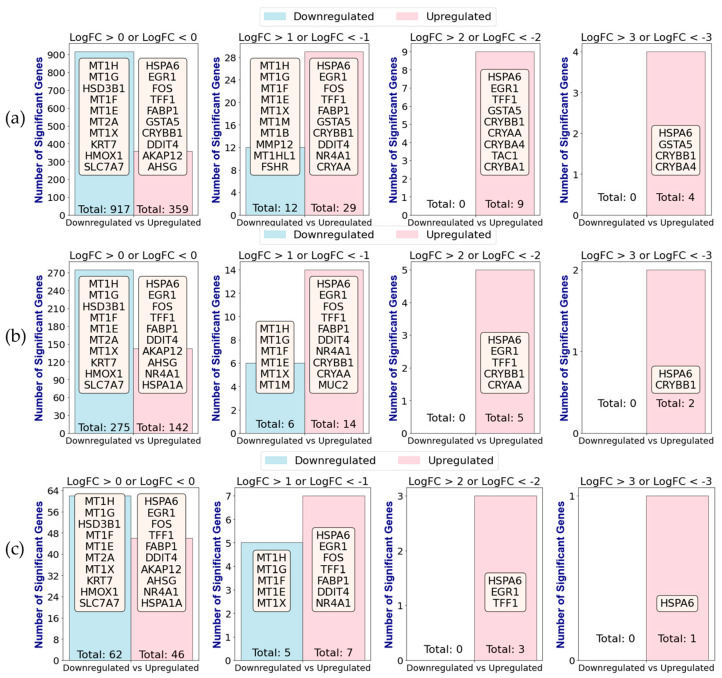
Comparison of significant gene selection across different statistical correction methods and LogFC thresholds after applying GLMQL-RMAS/MAS to contrast MPXV I-infected samples against mock (baseline) samples. Panels (**a**–**c**) illustrate the number and top 10 genes determined to be significant (**a**) using raw *p*-values, (**b**) Benjamini-Hochberg adjustment, and (**c**) Bonferroni correction, respectively. Each subpanel within (**a**–**c**) represents varying LogFC thresholds, from 0 to 3 for upregulated genes and from 0 to −3 for downregulated genes, highlighting the influence of statistical methodology and threshold settings on the identification of significant genes.

Moreover, RMAS/MAS ranking independently identifies top genes, regardless of log fold change (LogFC) thresholds or corrections for multiple hypothesis testing. After applying GLMQL to contrast MPXV I-infected samples against mock (baseline) samples, Figure 2 showcases the variability in the identification of top significant genes across three different statistical approaches: (a) using raw *p*-values (using RMAS for ranking), (b) applying the Benjamini-Hochberg (BH) method [33,34] for correction (using MAS for ranking), and (c) using the Bonferroni correction method [35] (using MAS for ranking). Each panel reflects gene significance at varying LogFC thresholds, ranging from 0 to 3 for upregulated genes and from 0 to −3 for downregulated genes. This selective ranking through RMAS and MAS is important as it narrows down the pool of input genes (features) for our machine learning models. The selection of top genes thereby becomes a key step in ensuring the robustness and accuracy of predictive models.

**Figure 3:**
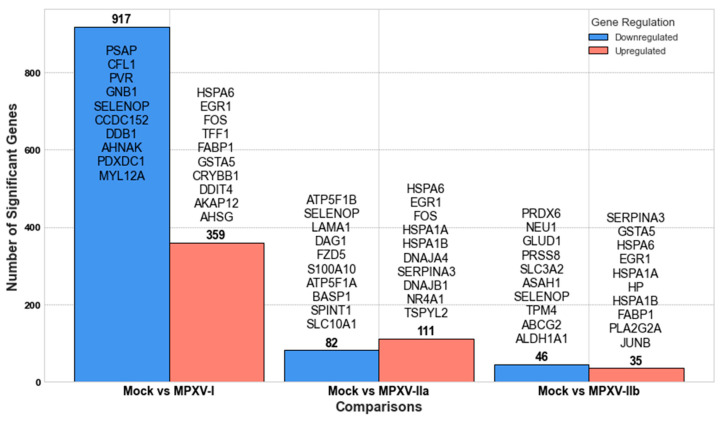
Gene expression analysis results using GLMQL-RMAS, identifying significant upregulated and downregulated genes across various MPXV clade comparisons with mock based on raw *p*-values. The genes are listed in descending order of their RMAS, where the top gene displays the maximum RMAS score (the maximum log fold change and the minimum *p*-value simultaneously), indicating the most significant expression difference in this analysis.

**Figure 4:**
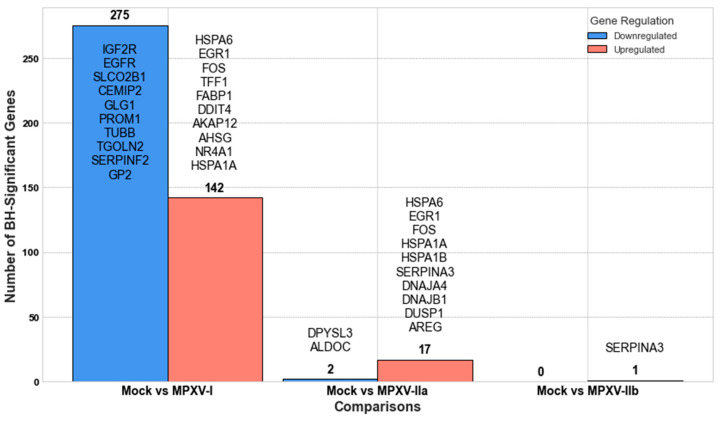
Differential gene expression analysis using GLMQL-MAS, with results adjusted using the Benjamini-Hochberg method. This figure displays significant upregulated and downregulated genes across different comparisons between mock and various MPXV clades. Each gene in the list is ranked according to its MAS score, with the first gene showing the highest differential expression based on the combined criteria of maximum LogFC and minimum BH-adjusted *p*-value.

Figure 3 and Figure 4 display the results from our GLMQL-RMAS and GLMQL-MAS analyses, respectively, across different comparisons between mock and various MPXV clades. Specifically, Figure 3 illustrates the significant upregulated and downregulated genes identified using raw *p*-values through GLMQL-RMAS, whereas Figure 4 shows those adjusted by the Benjamini-Hochberg method in the GLMQL-MAS analysis.

### 2.2. Comprehensive Gene Ontology (GO) Analysis

**Figure 5:**
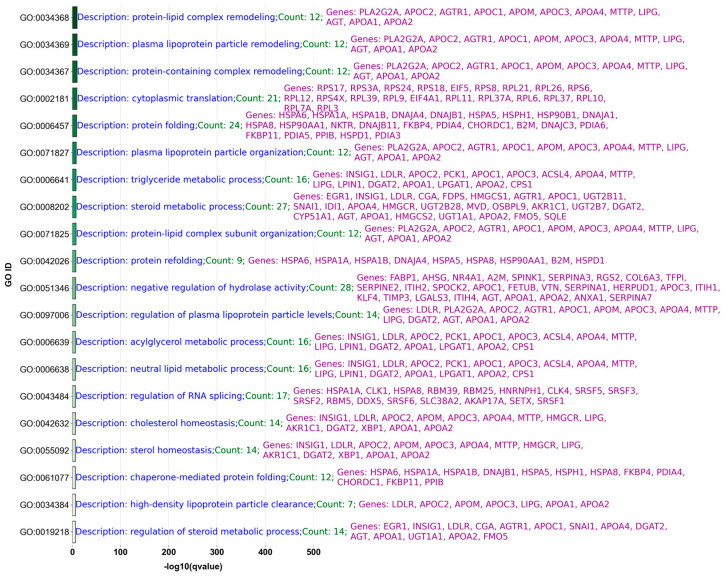
Top 20 GO biological processes associated with genes significantly upregulated in MPXV I compared to mock based on raw *p*-values. This representation includes genes deemed significant before the application of the Benjamini-Hochberg adjustment, allowing for a broader inclusion of differentially expressed genes.

Figure 5 displays the top 20 GO processes related to upregulated significant genes (based on raw *p*-value) identified from contrasting mock (baseline) vs. MPXV I samples. Figures S1 and S2, where “S” stands for Supplementary Material, present similar analyses for contrasts of mock vs. MPXV IIa and MPXV IIb samples, respectively, highlighting the biological processes most influenced by these viral clades.

### 2.3. Machine Learning Analysis Using Identified GLMQL-RMAS Genes

**Figure 6:**
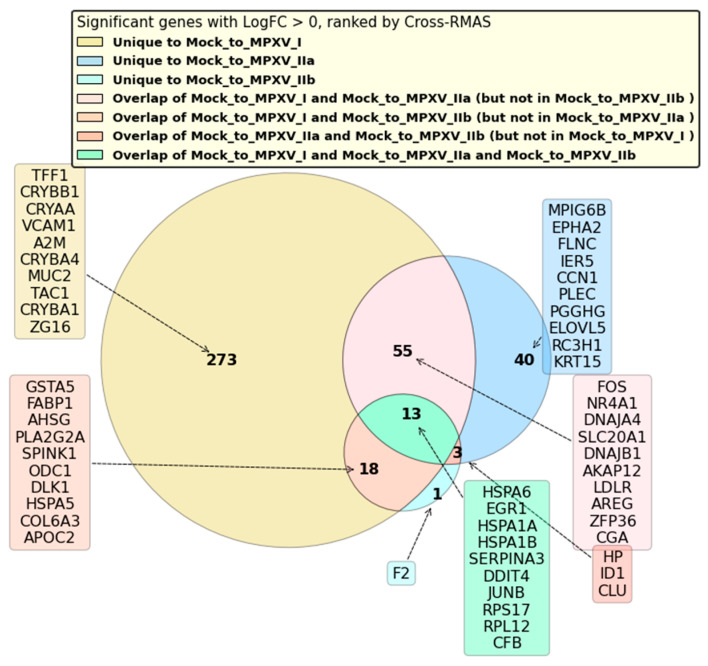
This Venn diagram (based on Cross-RMAS) illustrates the categorization of significantly upregulated GLMQL-RMAS-selected genes (LogFC > 0) based on their uniqueness or overlap among different contrasts (mock vs. MPXV I, mock vs. MPXV IIa, and mock vs. MPXV IIb). The diagram displays the seven groups of genes, ranked using the Cross-RMAS method. In this figure, genes are organized by their Cross-RMAS scores, which means that the first gene mentioned has the highest score (rank = 1), reflecting the strongest statistical and biological relevance among the displayed genes in each group.

**Figure 7:**
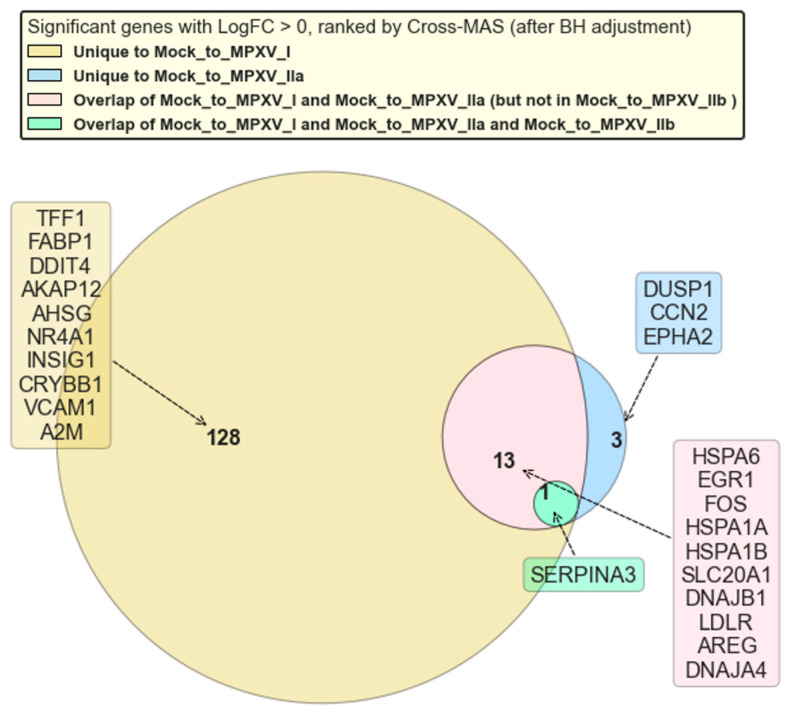
This venn diagram (based on Cross-MAS) illustrates the categorization of significantly upregulated GLMQL-MAS (using BH adjusted *p*-values) selected genes (LogFC > 0) based on their uniqueness or overlap among different contrasts (Mock vs. MPXV I, Mock vs. MPXV IIa, and Mock vs. MPXV IIb).

**Figure 8:**
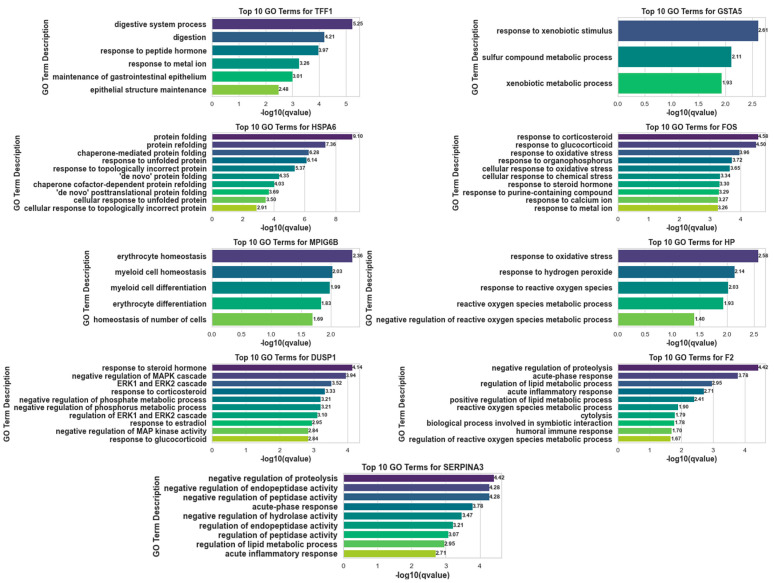
Using Gene Ontology (GO) terms derived from GLMQL-RMAS and GLMQL-MAS upregulated significant genes for the top Cross-RMAS and Cross-MAS selected genes for potential seven groups.

The categorization and Cross-RMAS-based ranking of significantly upregulated GLMQL-RMAS-selected genes are depicted in Figure 6. This figure illustrates the seven groups of genes based on their uniqueness or overlap among different contrasts (mock vs. MPXV I, mock vs. MPXV IIa, and mock vs. MPXV IIb). The top Cross-RMAS-selected genes for different groups include *TFF1*, unique to MPXV I; *MPIG6P*, unique to MPXV IIa; *F2*, unique to MPXV IIb; *FOS*, common between MPXV I and IIa; *GSTA5*, common between MPXV I and IIb; *HP*, common between MPXV IIa and IIb; and *HSPA6*, common to all clades. The categorization and ranking of significantly upregulated genes, selected by GLMQL-MAS and based on the Benjamini-Hochberg adjustment, are depicted in Figure 7. Figure 8 displays the top GO terms for the most significantly upregulated genes identified by Cross-RMAS and Cross-MAS.

**Figure 9:**
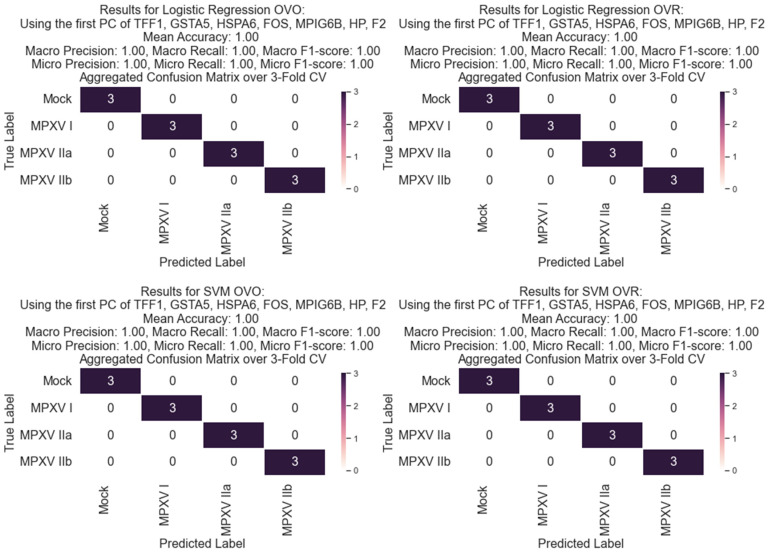
Confusion matrices depict the performance of Logistic Regression and SVM with a linear kernel using the first principal component of top genes identified by Cross-RMAS. These models use One-Versus-One (OVO) and One-Versus-Rest (OVR) strategies to differentiate between mock and various MPXV clades.

Figure 9 displays the confusion matrices of Logistic Regression (LR) [30,31] and Support Vector Machine (SVM) [32] with a linear kernel, evaluating performance using only the first principal component, PC1, derived from the top Cross-RMAS-identified genes for all unique and overlapping groups (see Figure 6). This figure indicates that both LR and the SVM with a linear kernel, employing both OVO and OVR strategies, achieves the highest performance with 100% accuracy in differentiating the mock and different clades from each other through a leave-one-out strategy [36] (3-fold stratified cross-validation [37], given that we have only three samples per condition).

**Figure 10:**
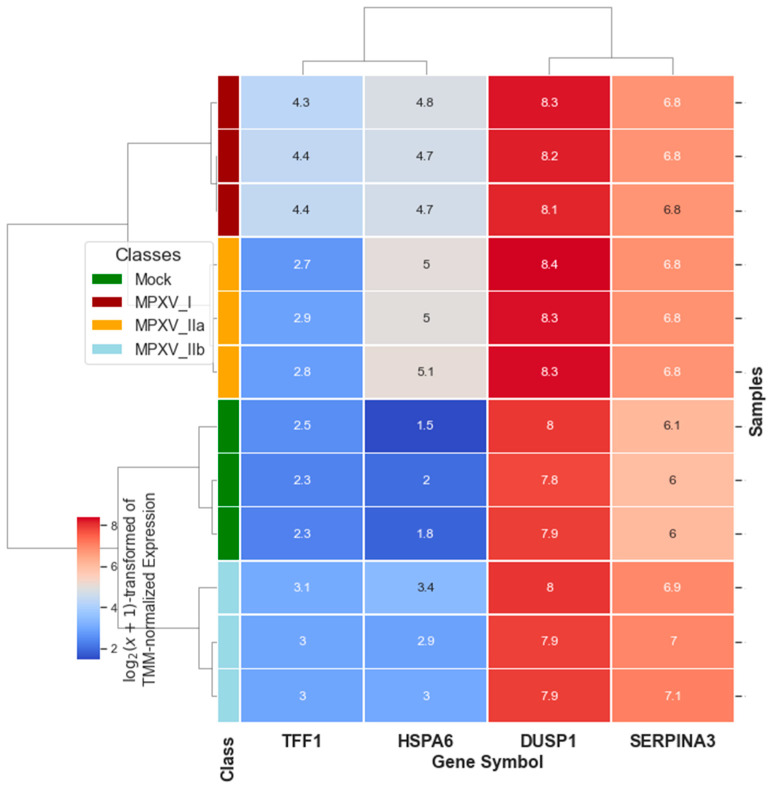
This heatmap shows the hierarchical clustering of samples using the top Cross-MAS-identified genes (*TFF1*, *HSPA6*, *DUSP1*, *SERPINA3*) within TMM normalized RNA-seq data. Clustering employed Euclidean distance and Ward’s linkage method, with data log2 transformed, adding a pseudocount of 1.

**Figure 11:**
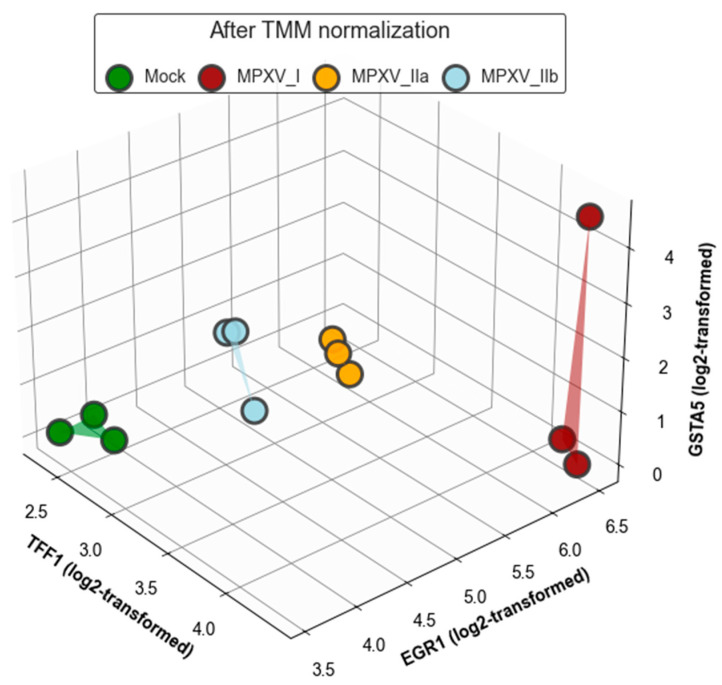
Three-dimensional visualization using *TFF1*, *EGR1*, and *GSTA5* as axes based on log2-transformed data with a pseudocount of 1.

Figure 10 shows a heatmap of hierarchical clustering using the top Cross-MAS selected genes (see Figure 7) within TMM normalized data. We employed a Euclidean distance metric and Ward’s linkage method, and the data were log2 transformed with a pseudocount of 1 to illustrate how clearly the samples were separated using only *TFF1*, *HSPA6*, *DUSP1*, and SERPINA3. Figure 11 presents a 3D visualization using *TFF1*, *EGR1*, and *GSTA5* as coordinates after log2 transformation with a pseudocount of 1, further demonstrating the distinct separation of the samples.

## 3. Discussion

### 3.1. GLMQL-RMAS: Generalized Linear Models with Quasi-Likelihood F-Tests and Relaxed Magnitude–Altitude Scoring

Figure 1 and Figure 2 collectively demonstrate the effectiveness of the RMAS and MAS ranking methods in identifying top genes from MPXV I-infected versus mock samples. Figure 1 shows that the genes ranked as most significant by RMAS are not only statistically robust but also biologically relevant. Figure 2 further illustrates that RMAS and MAS rankings are independent of different statistical correction methods, such as raw *p*-values, the Benjamini-Hochberg method, and the Bonferroni correction, as well as various LogFC thresholds. In all cases, *HSPA6* consistently emerged as the top upregulated significant gene, regardless of the statistical correction method or LogFC threshold.

Figure 3 presents the findings from the GLMQL-RMAS analysis, which identified DEGs in colon organoids infected with various MPXV strains. This analysis highlights a broad spectrum of gene expression alterations, offering insights into how the virus influences cellular pathways. Specifically, the comparison between the mock and MPXV I clade revealed remarkable changes with 359 upregulated and 917 downregulated significant genes. Notably, genes such as *HSPA6*, *EGR1*, *FOS*, and *TFF1* were upregulated, while downregulated genes like *PSAP*, *CFL1*, and *PVR* suggest suppression of processes crucial for vesicular trafficking, cytoskeletal organization, and cellular signaling, which are all vital for maintaining cellular integrity and communication.

Heat Shock Protein A6 (*HSPA6*), part of the Hsp70 family, primarily functions as a molecular chaperone [38], playing a key role in maintaining cellular homeostasis by assisting in the folding of nascent proteins and the refolding or degradation of misfolded proteins under stress conditions [39,40]. Although *HSPA6* does not directly interact with virion components, its general role in protein maintenance and stress responses can indirectly influence virion physiology [41]. During viral infections, the upregulation of heat shock proteins may assist in the proper folding and assembly of viral proteins, potentially facilitating virion assembly and impacting viral replication processes [42]. Additionally, *HSPA6*’s involvement in cellular stress responses, including its role in modulating the immune microenvironment in diseases like gliomas [43], suggests it may influence host defenses during viral infections, which could be pertinent to understanding interactions in viral pathologies such as MPXV.

Early Growth Response 1 (*EGR1*) is a zinc-finger transcription factor that plays an important role in the regulation of various cellular processes, including cell proliferation, differentiation, and apoptosis [44]. In the context of viral infections, *EGR1* is quickly activated and can influence host–pathogen interactions by modulating the expression of genes involved in immune responses [45]. For instance, during infections, such as with SARS-CoV-2, *EGR1* has been found to regulate the degradation of viral proteins, thus acting as a restriction factor and inhibiting viral replication [46]. Its rapid activation in response to stress and viral stimuli suggests that *EGR1* plays a role in modulating cellular stress responses, which are essential for both viral defense and pathogenesis [45]. *EGR1*’s activation during viral infections can regulate host proteins that degrade or misfold viral proteins, affecting viral particle assembly and replication [45].

The *FOS* gene, particularly through its protein product c-Fos (Proto-Oncogene c-Fos), plays a role in regulating gene expression in response to external stimuli, including viral infections [47]. While *FOS* does not directly contribute to virion physiology, its activation can significantly influence host cellular processes that are important for viral replication and immune responses [40]. Trefoil Factor 1 (*TFF1*) is predominantly recognized for its role in mucosal protection and repair in the gastrointestinal tract. It enhances epithelial healing and facilitates responses such as the epithelial-to-mesenchymal transition (EMT), particularly under hypoxic conditions, which are critical during cellular stress responses [48]. *TFF1* is also involved in innate immune defense by forming complexes with proteins like *FCGBP*, which bind to pathogens, indicating its role in microbial defense mechanisms [49]. Although *TFF1* is not directly linked to virion physiology, its involvement in maintaining mucosal integrity and facilitating immune defenses can indirectly influence virion dynamics, particularly in tissues susceptible to viral infections. The protein’s interactions and tumor suppressor functions in various cancers suggest broader biological roles that could intersect with viral pathology [50].

The expression profile for MPXV IIa (Figure 3) showed a notable difference, with 111 significant genes upregulated and 82 downregulated. The persistence of upregulated genes, such as *HSPA6* and *EGR1*, across different strains indicates a common stress response mechanism activated by MPXV infections. In contrast, the comparison involving MPXV IIb (Figure 3) showed fewer DEGs, with 35 upregulated and 46 downregulated significant genes, suggesting a potentially lower level of cellular disturbance or a more covert evasion strategy by this strain. The upregulation of genes, like *SERPINA3* and *GSTA5*, points to a defensive response by the host to mitigate protease activity and oxidative stress, which are likely induced by the viral infection.

These distinct patterns of gene expression across the three strains underscore the heterogeneity of MPXV pathogenesis and highlight the potential for strain-specific therapeutic targeting. MPXV I, showing the highest number of significant DEGs, suggests a strong and broad impact on the host cellular environment. The extensive modulation of host cell pathways may reflect a vigorous immune response and significant cellular reprogramming by the virus. In comparison, MPXV IIa demonstrates a markedly lower total number of DEGs, indicating less extensive alteration of host cellular functions. This could reflect a more streamlined or focused interaction with host cells, possibly revealing a different strategy for viral survival or evasion. MPXV IIb, showing the fewest significant DEGs, might indicate the least overall disruption of host cell functions among the three strains.

Following the application of the Benjamini-Hochberg method in our GLMQL-MAS analysis, Figure 4 presents a refined perspective on the impact of each MPXV strain on gene expression in colon organoids. These results in Figure 4 are compatible with those observed in Figure 3, though they reflect a more conservative interpretation of the data due to the stringent correction for multiple comparisons. Based on the number of significantly altered genes, MPXV I appears to be the most impactful strain, causing extensive changes in gene expression that could be associated with severe cellular and systemic responses. Conversely, MPXV IIb seems to be the least impactful in terms of DEGs, possibly indicating a more covert or less aggressive interaction with the host.

### 3.2. Comprehensive Gene Ontology (GO) Analysis

Our GO analysis for MPXV I, IIa, and IIb, represented (only the top 10 GO terms), respectively, in Figure 5, Figures S1 and S2, provides a detailed comparison of the upregulated significant genes and their associated GO terms, particularly focusing on their impact on colon-related processes. This analysis reveals important insights into the unique and common pathways that may influence the pathophysiology of the Mpox virus across different clades, thereby aiding in the development of targeted therapeutic and management strategies, particularly for gastrointestinal complications.

Starting with an overview of significant GO terms, MPXV I exhibits the highest number with 394 significant terms, followed by MPXV IIa with 178 and MPXV IIb with 151. Despite these differences, 33 GO terms are commonly identified across all clades, including those related to protein folding (GO:0006457), response to unfolded protein (GO:0006986), and chaperone-mediated protein folding (GO:0061077). These shared terms suggest a robust cellular response to the stress induced by the viral infection, which is critical for understanding how the virus manipulates host cellular machinery, especially within the colon where such stress responses could significantly influence gastrointestinal symptoms and outcomes.

In terms of unique attributes, MPXV I is distinguished by specific GO terms not shared with MPXV IIa and IIb, such as cellular response to chemical stimulus (GO:0070887) and the regulation of cellular amino acid metabolic process (GO:0006521). These unique terms may indicate clade-specific pathophysiological mechanisms that are more pronounced or exclusively present in MPXV I, potentially affecting how the virus interacts with the colon environment.

In the intricate landscape of Mpox’s impact on the colon, the GO terms associated with MPXV I offer valuable insights into a spectrum of processes that range from fundamental digestive functions to sophisticated immune responses within the gastrointestinal tract.

One important area involves the Digestive System Process (GO:0022600), where genes such as *TFF1*, *FABP1*, *MUC2*, *LDLR*, *TAC1*, *SERPINA3*, and *TFF2* play significant roles. *MUC2*, for example, encodes mucin, which is essential for forming a protective mucus barrier on the epithelial surface, safeguarding against pathogens. *TFF1* and *TFF2*, known as trefoil factors, contribute to mucosal healing and repair [51]. Disruptions in these gene functions could lead to compromised mucosal integrity, increasing susceptibility to secondary infections or inflammation [52] due to Mpox.

Furthermore, the terms Intestinal Absorption (GO:0050892) and related regulatory terms highlight the absorption of nutrients and cholesterol, which are crucial for maintaining energy balance. Genes like *LDLR* and *APOA1*, involved in lipid transport and metabolism, reflect how Mpox might alter metabolic processes in the colon, impacting nutritional status and immune responses. *LDLR* is known for its role in cholesterol metabolism, which is very important for cell membrane integrity and function, potentially affecting how the colon responds to and recovers from infectious diseases [53], like Mpox. *APOA1*, the primary protein component of high-density lipoprotein (HDL), plays a key role in the reverse cholesterol transport pathway, which is essential for removing cholesterol from tissues and promoting anti-inflammatory effects in the body [54].

The Maintenance and Structure of Gastrointestinal Epithelium (GO:0030277, GO:0010669) involves genes such as *MUC2*, *SERPINA3*, *TFF1*, *TFF2*, and *RBP4*, which are important in maintaining epithelial integrity and preventing pathogen invasion [55].

Moreover, the Immune Response in the Gastrointestinal Context (GO:0002286, GO:0002683, GO:0002697, GO:0002698, GO:0002699) encompasses genes like *A2M*, *PCK1*, *NFKBIZ*, *IL4R*, *LGALS3*, *HLA-B*, and *CEACAM1*, which are integral to T cell activation and immune regulation [56,57]. For instance, *IL4R*’s role in cytokine signaling [57] and *HLA-B*’s involvement in antigen presentation [58] highlight the crucial aspects of immune response modulation during Mpox infection, which can significantly influence disease severity and progression.

This comprehensive GO analysis underscores the influence of Mpox on various biological processes within the colon, from nutrient absorption and immune responses to maintaining epithelial integrity. The genes and GO terms discussed not only provide a deeper understanding of the pathogenic mechanisms but also point towards potential therapeutic targets and biomarkers essential for assessing the impact of Mpox on gastrointestinal health.

### 3.3. Machine Learning Analysis Using Identified GLMQL-RMAS Genes

The analysis depicted in Figure 6 showcases the differential gene expression impacted by various MPXV clades, illustrating the interplay of biological responses that are both unique to each clade and common across multiple ones. Specifically, the Venn diagram indicates that 273 genes are uniquely upregulated in response to the Mock (baseline) versus MPXV I contrast, signaling a distinct genomic response to this clade. This unique expression might suggest specific viral interactions or pathogenic mechanisms exclusive to MPXV I. Additionally, the responses to MPXV IIa and MPXV IIb contrasts are notably different, with 40 genes and just 1 gene uniquely upregulated, respectively, indicating more focused genomic activations that could be targets for clade-specific medical interventions or diagnostic efforts.

Moreover, the diagram also identifies genes that respond across multiple clade contrasts but not for all. There are 55 genes commonly upregulated in responses to MPXV I and MPXV IIa but absent in MPXV IIb, 18 genes common to MPXV I and MPXV IIb but not MPXV IIa, and 3 genes upregulated in both MPXV IIa and MPXV IIb, yet not in MPXV I. Importantly, the presence of 13 genes upregulated across all three clades highlights pathways in the host response that are conserved, regardless of the viral clade, emphasizing their potential as targets for broad-spectrum therapies. 

Figure 7 refines the analysis by focusing on genes that meet the BH-significance criteria for their corresponding contrasts, providing a more stringent insight into clade-specific gene expression. This figure reveals that 128 genes are uniquely upregulated in response to MPXV I, showcasing a significant and distinct genomic reaction to this clade. In contrast, only 3 genes are uniquely upregulated in response to MPXV IIa, while no genes are uniquely upregulated in response to MPXV IIb, highlighting the variability in host response to different clades. Additionally, the figure illustrates that *SERPINA3* is the only gene significantly upregulated across all clades, suggesting its pivotal role in the host’s defense mechanism against MPXV infection. Moreover, 13 genes are significantly upregulated in response to both MPXV I and MPXV IIa, but not MPXV IIb, indicating shared biological pathways between these two clades that are not activated in response to MPXV IIb.

In the analysis presented in Figure 8, the top GO terms associated with the genes identified through Cross-RMAS (Figure 6) and Cross-MAS (Figure 7), provide significant insights into their biological functions and implications for Mpox infection. The GO terms for *TFF1* emphasize its role in the gastrointestinal system, which is directly relevant to the clinical observations of Mpox-associated GI manifestations. Notably, terms such as “digestive system process” and “maintenance of gastrointestinal epithelium” suggest *TFF1*’s involvement in maintaining the structural and functional integrity of the GI tract. These processes are vital in understanding how Mpox may disrupt normal digestive functions and epithelial barriers, contributing to symptoms like proctitis and abdominal pain observed in patients. *GSTA5*, highlighted in Figure 8, is associated with “response to xenobiotic stimulus” and “xenobiotic metabolic process”. These terms suggest *GSTA5*’s role in the detoxification pathways, which may be activated in response to viral infection to handle the increased load of foreign molecules. This is critical for mitigating the cellular stress and damage induced by the viral invasion, potentially reducing the severity of the infection’s impact.

*HSPA6*’s involvement in protein management processes, such as “protein folding” and “response to unfolded protein”, as shown in Figure 8, is particularly important under the stress of viral infection. *HSPA6* helps to manage the increased demand for folding new viral and cellular proteins, ensuring cellular homeostasis and reducing misfolded proteins that can lead to cellular dysfunction. The GO terms associated with *FOS* involve various stress responses, including “response to corticosteroid” and “response to oxidative stress”, emphasizing its role in modulating the host’s defense mechanisms. The expression of *FOS* during oxidative stress is indicative of its function in signaling pathways that activate inflammatory and immune responses, which are crucial for combating viral infections like Mpox.

Shifting the focus to *TFF1*, the top GLMQL-RMAS-selected unique gene for MPXV I, this gene plays an important role in maintaining epithelial integrity and is primarily expressed in the gastric epithelium and, to a lesser extent, across the mucosal surfaces of the gastrointestinal tract [59]. *TFF1* is very important for the stabilization of mucous gels, providing protection against mechanical damage, chemical irritants, and pathogens that threaten the lining of the stomach and intestines [49].

In the realm of mucosal healing, *TFF1* is notably involved in mucosal protection and repair [49]. It promotes cell migration and epithelial restitution, crucially without proliferative changes [51]. This function becomes particularly significant in the context of gastrointestinal diseases or conditions that involve mucosal damage, such as ulcers or inflammatory responses triggered by infections [51].

Moreover, *TFF1* possesses anti-apoptotic properties, aiding in the prevention of programmed cell death in epithelial cells. This capability is essential for maintaining cell integrity under stress conditions, such as those induced by infections or inflammatory reactions [60]. During Mpox infection, the role of *TFF1* becomes even more pertinent. The integrity of mucosal barriers can be compromised, heightening susceptibility to secondary infections and exacerbating inflammation. *TFF1*’s role in fortifying mucosal defenses is important [52], suggesting a protective role against the spread and severity of the infection within the gastrointestinal tract.

Furthermore, given that Mpox can cause lesions and other mucosal disruptions [1], *TFF1*’s involvement in promoting rapid healing of the epithelium could significantly reduce the duration and severity of such manifestations. Its ability to enhance epithelial repair without promoting excessive cell proliferation [52] makes it a key factor in maintaining normal gastrointestinal function during and after infection.

For MPXV IIa, top GO terms associated with *MPIG6B* like “erythrocyte homeostasis” and “myeloid cell homeostasis” emphasize roles in regulating blood cell stability and immune cell equilibrium, which might be important during viral infections to maintain systemic balance. Similarly, the involvement of *HP* in “response to oxidative stress” and “response to reactive oxygen species” indicates its role in mitigating oxidative damage during infections, enhancing cellular resilience against viral onslaughts. *DUSP1*, with its multiple roles in modulating the MAPK cascade and response to steroid hormones, points to its involvement in signaling pathways that regulate inflammation and immune responses, potentially influencing the host’s defensive mechanisms against the viral infection.

In contrast, for MPXV IIb, the gene *F2* is associated with “acute-phase response” and “regulation of lipid metabolic process”, reflecting its significant involvement in the immediate immunological response to infection and metabolic adaptations. *SERPINA3*, sharing similar GO terms with *F2*, also underscores its role in the negative regulation of proteolytic processes and acute-phase responses, which are important in controlling inflammation and preventing excessive protease activity that could damage host tissues during viral infections. 

Figure 9 demonstrates the effectiveness of machine learning models in differentiating between different strains of Mpox and mock samples using the Cross-RMAS-selected genes: *TFF1*, *GSTA5*, *HSPA6*, *FOS*, *MPIG6B*, *F2* and *HP*. These genes were identified as key genes capable of distinguishing between Mock and various Mpox strains, emphasizing their significant roles in the pathophysiological processes of the virus.

The LR and SVM models, using only the first principal component derived from these genes, showcased a robust capability to classify between the different strains and mock samples. Both the One-Versus-One (OVO) and One-Versus-Rest (OVR) strategies were employed, with the LR and the SVM models achieving perfect (with 100% accuracy) classification metrics across all categories, demonstrating a mean accuracy, macro precision, recall, and F1-score of 100% in both OVO and OVR setups.

Note that we took all necessary precautions to mitigate the risk of overfitting, given our limited sample size. We employed linear classifiers, Logistic Regression and SVM with a linear kernel, which are inherently less prone to overfitting due to their simplicity. Additionally, we focused on dimensionality reduction using only the first principal component derived from the key genes. To further ensure the robustness and generalizability of our models, we implemented 3-fold stratified cross-validation across our data. These strategic measures collectively enhance the reliability and validity of our classification outcomes.

The importance of these results lies in the validation of *TFF1*, *GSTA5*, *HSPA6*, *FOS*, *MPIG6B*, *F2* and *HP* not only as key markers for the presence of infection but also for their specificity in differentiating between various Mpox strains. This capability is important, especially given that the ability to distinguish between different strains can significantly enhance our understanding of the epidemiological dynamics of the virus and inform targeted public health responses and treatment strategies.

Moreover, these results underscore the potential of these genes as powerful tools for diagnostic purposes. While the top GLMQL-RMAS-selected genes can also achieve high accuracy in distinguishing specific strains from mock in their respective contrasts, the use of Cross-RMAS-selected genes extends this capability across multiple strains, offering a broader application for surveillance and diagnosis. Figure 10 presents a heatmap displaying the expression levels of the Cross-MAS-selected genes, *TFF1*, *HSPA6*, *DUSP1* and *SERPINA3*, across different Mpox strains (MPXV I, MPXV IIa, MPXV IIb) and mock samples. This visualization clearly demonstrates the distinct expression profiles of these genes, which correspond to the specific viral strains, showing clean clustering and separation of the different classes.

Figure 11 presents the 3D visualization of the distinct separation among mock, MPXV I, MPXV IIa, and MPXV IIb samples based on the expression levels of three genes, *TFF1*, *EGR1*, and *GSTA5*, after TMM normalization. This graphical representation clearly illustrates the effective differentiation of each strain and the mock samples using the selected biomarkers. This distinct pattern not only confirms the specificity of the response to each strain but also underscores the potential of these genes as diagnostic markers capable of distinguishing between closely related viral strains.

In summary, our analysis highlights distinct gene expression patterns across different MPXV clades, showcasing how these strains uniquely affect host cellular functions. Clade I of MPXV exhibits significant changes in gene expression, with notable upregulation of stress response genes like *HSPA6*, *EGR1*, *FOS*, and *TFF1*, and downregulation of genes such as *PSAP* and *CFL1* that are important for vesicular trafficking and cytoskeletal organization. In contrast, MPXV Clades IIa and IIb display fewer changes, indicating a more contained interaction with host cells. Despite fewer differentially expressed genes, Clade IIa shares stress response elements with Clade I, notably in the upregulation of *HSPA6* and *EGR1*, suggesting a universal host response mechanism. Clade IIb shows minimal gene expression changes, potentially indicating efficient evasion of host defenses or less aggressive pathogenicity, with specific upregulation of *SERPINA3* and *GSTA5* highlighting targeted defense mechanisms against protease activity and oxidative stress.

Furthermore, the outstanding classification accuracy achieved by machine learning models using these genes underscores their utility not just as markers of infection but as specific indicators capable of differentiating between Mpox strains. This capability is important for advancing our understanding of the virus’s epidemiological dynamics and for developing more precise public health strategies and therapeutic interventions. Table 1 compares the overall findings and methodologies between our study and those of Watanabe et al. [13], summarizing the distinct approaches and insights each study brings to the understanding of MPXV infections in human-derived cellular models.

## 4. Materials and Methods

Watanabe et al. [13] developed colon organoids from human-induced pluripotent stem cells (iPSCs) to investigate the infectivity of different MPXV strains. The iPSC line, 1383D6, was cultured on recombinant human laminin and subjected to various differentiation protocols to simulate colon organoid development [13]. Initially, iPSCs underwent definitive endoderm and hindgut differentiation using a combination of growth factors and inhibitors. The cells were then embedded in a growth factor-reduced Matrigel to form colon organoids. For colonic differentiation, a combination of CHIR99021, A-83-01, Noggin, Forskolin, and EGF was used [13].

The organoids were extracted from the Matrigel and seeded onto Matrigel-coated plates [13]. They were then exposed to three MPXV strains: Zr-599 (Congo Basin strain, MPXV I), Liberia (West African strain, MPXV IIa), and the 2022 outbreak strain (MPXV IIb). The virus was prepared using VeroE6 cells, and infectious titers were determined by plaque assays [13]. The impact of MPXV on the organoids was analyzed by quantifying viral DNA and assessing changes in mRNA expression levels through real-time PCR and RNA sequencing [13]. This comprehensive approach allowed Watanabe et al. [13] to closely mimic the infection dynamics in human colonic tissue, providing valuable insights into the pathogenesis and potential treatment strategies for MPXV infections.

For our analysis, we accessed and used the RNA-seq count data from the colon organoid samples infected with each MPXV clade, available under GEO accession number GSE219036, as described in Watanabe et al. [13]. Next, we refined the gene expression data by mapping the Gene IDs to their corresponding gene symbols using the MyGene.info API (https://mygene.info/) [61]. In this study, we have specifically focused our RNA-seq analysis on protein-coding genes, owing to their direct involvement in cellular functions and disease mechanisms. By targeting these genes, we aimed to elucidate the roles they play in the biological pathways affected by the conditions under study, thereby offering more actionable insights for therapeutic interventions. To enhance the reliability and accuracy of our gene expression analysis, we integrate appropriate statistical methodologies and normalization techniques.

### 4.1. GLMQL-RMAS: Generalized Linear Models with Quasi-Likelihood F-Tests and Relaxed Magnitude–Altitude Scoring

In this section, we have used proper statistical methodologies and normalization techniques to enhance the reliability and accuracy of our RNA-seq gene expression analysis, focusing on samples exposed to different strains of the Mpox virus: MPXV I, MPXV IIa, and MPXV IIb, with mock serving as the baseline control. Our goal was to identify differentially expressed genes (DEGs) with high confidence, employing stringent criteria for statistical significance. Recognizing the critical role of normalization in RNA-seq data analysis, we initiated the analysis by employing TMM normalization [18] to correct library-specific compositional differences.

Given the inherent challenges of RNA-seq data, such as their non-normal distribution and common overdispersion where variance exceeds the mean [62], we opted to use Generalized Linear Models (GLMs) [23] tailored for RNA-seq count data. These models, appropriate for the discrete nature of count data, typically follow negative binomial distributions. For each experimental setup, contrasting mock against each Mpox strain, we defined experimental groups, each with three replicates, to maintain statistical robustness. The mock condition was designated as the reference level against which all other conditions were compared, a crucial setup for isolating the genetic impacts specific to each virus strain.

We constructed a design matrix for each comparison, incorporating all conditions. This matrix was crucial for modeling the influence of experimental variables on gene expression. A Generalized Linear Model was then fitted to the TMM-normalized data, and dispersion estimates were calculated to inform the model about the variability within the data, allowing for more accurate assessments of differential expressions.

Following the model fitting, Quasi-Likelihood F-tests [24] were performed to compare the full model, which included both the control and one of the virus strains, against a reduced model that excluded the virus strain. This step was facilitated by specifying the coefficient for the desired contrast, which directly addresses the changes attributed to the virus exposure compared to the baseline. The flexibility of Quasi-Likelihood F-tests is particularly advantageous for RNA-seq data, accommodating the unique distribution characteristics of the data without the strict assumptions required by traditional parametric tests. Next, for each gene, the estimated log fold change (LogFC) and the associated *p*-values were calculated. Adjusted *p*-values were applied using the Benjamini-Hochberg [33,34] method, with a significance level threshold of α=0.05, ensuring that only genes with statistically significant were considered differentially expressed.

Finally, we have incorporated the Relaxed Magnitude–Altitude Scoring (RMAS) methodology, which is a variant of Magnitude–Altitude Scoring (MAS) [19,20,21,22], into our analysis to enhance the identification and prioritization of differentially expressed genes. MAS method combines the absolute value of the LogFC (${|{log}_{2}(\mathrm{FC}}_{l})|$) with the Benjamini-Hochberg-adjusted *p*-value ($\left|{{log}_{10}(p}_{l}^{BH})\right|$) for each BH-significant gene $g_{l}$. This scoring system, expressed as ${MAS}_{l}={|{log}_{2}(\mathrm{FC}}_{l}{)|}^{M}|{{log}_{10}(p}_{l}^{BH}){|}^{A}$, where $p_{l}^{BH}$ denotes Benjamini-Hochberg-adjusted *p*-values, allows us to assess both the magnitude of expression changes and their statistical significance. Here, M and A are hyperparameters that fine tune the balance, ensuring a comprehensive evaluation of each gene’s relevance (in this study, we set M=A=1). This approach is detailed in our previous studies [19,20,21,22].

However, with only a few (three) samples per condition, the likelihood of identifying significantly differentially expressed genes using false discovery rate corrections, like the Benjamini-Hochberg (BH) method, is considerably reduced. The BH method controls the false discovery rate by adjusting *p*-values, which can become overly stringent when applied to small datasets. With fewer data points, the statistical power to detect true effects is limited, often resulting in higher adjusted *p*-values that do not meet the threshold for significance. Consequently, even potentially relevant biological signals can be deemed nonsignificant, suppressing meaningful findings. To counteract this limitation and avoid overlooking biologically significant genes, we employ the RMAS method. RMAS uses the unadjusted *p*-values ($p_{l})$ in place of BH-adjusted ones ($p_{l}^{BH})$ and is defined as ${RMAS}_{l}={|{log}_{2}(\mathrm{FC}}_{l}{)|}^{M}|{log}_{10}(p_{l}){|}^{A}$. This adaptation allows us to consider genes that may be biologically significant but overlooked due to stringent statistical thresholds, expanding our analytical scope as previously explored in our research [19].

In our analysis, the ranking system based on RMAS and MAS scores plays a crucial role not only in highlighting the immediate statistical and biological relevance of genes but also serves as a foundational element for more complex evaluations. Specifically, in Section 4.3, we use the rankings established here, where the gene signature most closely related to the MPXV infection and possessing the highest RMAS or MAS score is assigned rank 1, representing the smallest rank number. This rank is then used to define the Cross-RMAS rank, clearly indicating that the smallest rank, and thus the largest score, identifies the gene signature most pertinent to the MPXV infection. Algorithm 1 provides an overview of the GLMQL-RMAS process.
**Algorithm 1.** Differential Expression Analysis using GLMQL-RMAS**Input** : RNASeq count data for a control group with m samples and a treated group with n samples.**Output**: Table of genes with corresponding *p*-values, log fold changes, and RMAS scores.**Step 1:** Read count data from CSV file:
     data←read.csv(“Path_to_file.csv”, header=TRUE, row.names=1)
**Step 2:** Define conditions for each sample:
     conditions←c(rep(“Control”, times=m), rep(“Treated”, times=n))
**Step 3**: Initialize DGEList object:
     $dge\leftarrow DGEList\left(counts=data,group=conditions\right)$
**Step 4**: Normalize data using TMM normalization:
     $dge\leftarrow calcNormFactors\left(dge\right)$
**Step 5**: Create a design matrix for the model:
     $design\leftarrow model.matrix\left(\sim conditions\right)$
**Step 6**: Estimate dispersion:
     $dge\leftarrow estimateDisp\left(dge,design\right)$
**Step 7**: Fit GLM with quasi-likelihood:
     $fit\leftarrow glmQLFit\left(dge,design\right)$
**Step 8**: Perform quasi-likelihood F-test:
     $qlf\leftarrow glmQLFTest\left(fit,coef=2\right)$
**Step 9**: Get top differentially expressed genes:
     $results\leftarrow topTags\left(qlf,n=Inf\right)$
**Step 10**: Compute RMAS for each gene:   **for**
gene in results.table **do**
     gene$RMAS← abs(log10(gene$PValue))×abs(log2(gene$logFC))

   **end for**
**Step 11**: Sort genes by RMAS in descending order:
     results.table←results.table[order(−results.table$RMAS),]
**Step 12**: Format results with gene symbols:
     results.table←cbind(“Gene Symbol”=rownames(results$table), results$table)
**Step 13**: Output results to a CSV file:
     $file\_name\leftarrow paste0\left(“\mathrm{GLMQL}\text{\_}\mathrm{Results}.\mathrm{csv}”\right)$

     $write.csv\left(as.data.frame\left(results.table\right),file=file\_name,row.names=FALSE\right)$
**End (Algorithm 1)**

### 4.2. Comprehensive Gene Ontology (GO) Analysis

In this section, we aimed to delineate the biological processes influenced by gene expression changes following MPXV infection. This was achieved by focusing separately on upregulated and downregulated genes, identified through our enhanced GLMQL-RMAS analytical framework. For each group, GO enrichment was performed, focusing on biological processes, cellular components, and molecular functions. This analysis used the clusterProfiler [29] package within R, playing a key role in categorizing the identified genes into groups associated with these categories. By mapping our significant genes to specific GO terms, we illuminated the functional characteristics of these genes, thereby providing a detailed view of their roles within the cellular environment.

### 4.3. Machine Learning Analysis Using Identified GLMQL-RMAS Genes

In this section, we used machine learning techniques to differentiate between various strains of the MPXV and mock samples. We identify key genes using the GLMQL-RMAS approach. Our analytical framework employs Logistic Regression (LR) [30,31] and Support Vector Machine (SVM) [33] using both One-Versus-One (OVO) and One-Versus-Rest (OVR) strategies. Due to the limited size of our dataset, which includes only three samples per condition, we use k-fold stratified cross-validation (k = 3) [37] or leave-one-out cross-validation [36] to maximize the contribution of each sample to both training and validation, preserving the integrity of our model evaluations.

The primary challenge of employing machine learning models with small sample sizes is the high risk of overfitting [63]. Overfitting occurs when a model learns the noise and anomalies in the data as significant patterns due to a disproportionately high number of parameters relative to the available training data. This often results in models that perform well on training data but poorly on unseen data, failing to generalize effectively. To mitigate overfitting and enhance the robustness of our models, we deliberately limit the number of input features (genes). In our study, we employ a focused approach to mitigate the risk of overfitting associated with using thousands of genes. We specifically target significantly upregulated GLMQL-RMAS-selected genes from contrasts between mock (baseline) and each MPXV strain (MPXV I, MPXV IIa, and MPXV IIb) sample because these genes are most likely to reflect active biological processes and pathogenic mechanisms in response to the virus. Upregulated genes provide crucial insights into the host’s response to infection and are potentially more relevant for identifying targets for therapeutic interventions.

Since our goal is to identify genes capable of differentiating between different clades and the mock, once identified, these significant GLMQL-RMAS upregulated genes are categorized into potentially seven distinct groups based on whether they are unique to one contrast or overlap among multiple contrasts: unique to MPXV I, unique to MPXV IIa, unique to MPXV IIb, common to MPXV I and MPXV IIa, common to MPXV I and MPXV IIb, common to MPXV IIa and MPXV IIb, and common to all MPXV strains. This categorization enables a detailed analysis by highlighting the specific or shared roles of genes across different viral strains. To effectively prioritize these genes, we apply a cross-ranking method for overlapping and unique genes, called Cross-RMAS, which aggregates and compares RMAS ranks across relevant contrasts and ranks genes accordingly. The Cross-RMAS process for three contrasts (mock vs. three MPXV clades) is as follows:

**For Genes Unique to Each Contrast.** Genes identified by GLMQL-RMAS as upregulated and significant exclusively within a specific contrast, e.g., mock (baseline) vs. MPXV I, are considered unique to that contrast. These genes are ranked based on their RMAS rank within the entire set of GLMQL-RMAS-selected genes for that contrast.

**For Overlapping Groups of Two Contrasts.** For genes identified as GLMQL-RMAS significant in any two of the three contrasts, we rank them based on their RMAS rank in both contrasts. This process involves filtering and combining the gene RMAS ranks from both contrasts. Specifically, for all significant genes, we compare the RMAS ranks for each gene across the two contrasts, assigning the larger RMAS rank to each gene as its maximum rank. We then rank the genes based on these maximum RMAS ranks. The gene with the smallest of these maximum RMAS ranks across the two contrasts is assigned rank 1. This method effectively prioritizes genes that demonstrate the highest consistent significance across the two contrasts, ensuring that the most relevant genes are identified and appropriately ranked.

**For Overlapping Groups of Three Contrasts.** Genes selected by GLMQL-RMAS for all three contrasts (mock vs. MPXV I, mock vs. MPXV IIa, and mock vs. MPXV IIb) are ranked based on their RMAS ranks across all three contrasts. A function filters and combines these genes’ RMAS ranks into a single dataset, identifying the maximum RMAS rank for each gene across the contrasts. The gene with the smallest of these maximum ranks is selected, emphasizing those that consistently exhibit high relevance across all comparisons. Algorithm 2 provides an overview of the Cross-RMAS process.
**Algorithm 2.** Cross-RMAS**Input**: Dataframes of significant genes from contrasting three clades of MPXV against Mock, each with ordered RMAS scores, obtained from GLMQL-RMAS.**Output**: Categorized and ranked genes among all seven possible unique and common groups across contrasts.**Step 1.** Initialize lists to categorize genes based on their expression profiles across contrasts: Unique-I, Unique-IIa, Unique-IIb, Common-I-IIa, Common-I-IIb, Common-IIa-IIb, Common-All.**Step 2.** Analysis for each gene from the input dataframes:     **for** gene in each dataframe of significant genes, evaluate the presence of the gene across contrasts:        **If** gene is found only in one contrast dataframe then         ▪Add gene to the corresponding unique list.         ▪Assign Rank to gene based on its RMAS rank within that contrast.        **If** gene is found in exactly two contrast dataframes then         ▪Determine the maximum RMAS rank from those two contrasts.         ▪Add gene to the corresponding two-contrast common list.         ▪Assign Rank to gene based on the determined maximum RMAS rank.        **If** gene is found in all three contrast dataframes then         ▪Determine the maximum RMAS rank across all three contrasts.         ▪Add gene to the corresponding list: Common-All.         ▪Assign Rank to gene based on the determined maximum RMAS rank.**Step 3**: For each list (unique and common), sort genes by assigned rank in ascending order.**Step 4**. Input these genes (or perform PCA for dimensionality reduction) into machine learning models.**End (Algorithm 2)**

Cross-RMAS strategically selects genes that exhibit the maximum log fold change (LogFC) and the minimum *p*-value across all three contrasts simultaneously, ensuring the identification of genes with the most statistically significant changes in expression. Including all top Cross-RMAS-selected genes from the seven potential groups as inputs for the machine learning model ensures a comprehensive analysis of gene expression patterns across different conditions. Each group represents distinct or overlapping genetic responses to various Mpox virus strains, capturing both unique and shared gene functions. However, due to the limited number of samples, we use the first three principal components (PCs) [64] derived from the top Cross-RMAS-selected genes of these groups to effectively reduce dimensionality while retaining the most informative variance in the data. By incorporating these PCs, we maximize the model’s ability to distinguish between mock and infected samples by leveraging the full spectrum of genetic diversity, enhancing the model’s sensitivity to detect subtle differences. This approach robustly characterizes the biological mechanisms underlying each strain’s impact and ensures the model can more accurately classify samples based on intricate gene expression profiles, leading to more precise predictions and a better understanding of the genetic factors involved in Mpox virus infection. Note that if we apply GLMQL-MAS, which involves applying BH-significant adjustment instead of using raw *p*-values, then Cross-RMAS becomes Cross-MAS.

## 5. Conclusions

This study presented a comprehensive analysis of the Mpox virus’s impact on the colon organoids, leveraging appropriate methodologies, such as GLMQL-RMAS (Generalized Linear Models with Quasi-Likelihood F-Tests and Relaxed Magnitude–Altitude Scoring) and Cross-RMAS. By employing these methods, we have demonstrated their efficacy in overcoming the limitations of traditional statistical methods for RNA-seq analysis. GLMQL-RMAS, in particular, has proven to be a powerful tool for identifying DEGs with high confidence, considering both statistical significance and biological relevance, even in the presence of non-normal distribution and overdispersion typical of RNA-seq datasets.

Through our analysis, we identified several key genes, such as *TFF1*, *GSTA5*, *HSPA6*, *FOS*, *MPIG6B*, *F2*, *HP*, *DUSP1* and *SERPINA3*, which are not only significantly upregulated in response to Mpox infection but also serve as key genes for distinguishing between different viral clades. These genes have been further validated through Cross-RMAS ranking, which provides a robust framework for prioritizing genes based on their relevance across multiple conditions. The ability of these methods to identify strain-specific gene expression profiles underscores their potential for advancing our understanding of viral pathogenesis and for developing targeted therapeutic strategies.

Biologically, our findings offer novel insights into the pathophysiological processes triggered by different Mpox virus strains. The differential expression patterns observed among MPXV Clades I, IIa, and IIb suggest distinct mechanisms of host–pathogen interaction, with MPXV I causing the most extensive gene expression changes, indicative of a broad and aggressive impact on host cellular pathways. In contrast, MPXV IIb appears to adopt a more covert strategy, potentially minimizing detection by the host immune system or causing less disruption to host cellular processes.

The GO analysis has further illuminated the specific biological processes disrupted by Mpox infection. For instance, the upregulation of genes involved in protein folding and stress responses, such as *HSPA6*, indicates a heightened cellular effort to manage the increased protein synthesis and folding demands imposed by the virus. This response is particularly crucial in the colon, where maintaining epithelial integrity and function is vital for preventing secondary infections and managing gastrointestinal complications.

Moreover, the identification of genes like *TFF1*, which is crucial for maintaining mucosal integrity, highlights potential therapeutic targets for mitigating the gastrointestinal manifestations of Mpox. *TFF1*’s role in epithelial repair and protection against mucosal damage could be very important in reducing the severity of gastrointestinal symptoms, such as proctitis and abdominal pain, which are commonly observed in infected patients.

The machine learning models built using GLMQL-RMAS-identified genes have demonstrated 100% accuracy in distinguishing between different Mpox strains and mock samples. This capability is important for improving diagnostic precision and understanding the epidemiological dynamics of the virus. By effectively classifying samples based on specific gene expression profiles, these models provide a powerful tool for real-time monitoring and response to Mpox outbreaks.

### Limitations of Study

While this study provides valuable insights into the impact of the Mpox virus on colon organoids, several limitations must be acknowledged. First, the use of colon organoids as a model system, while advantageous for mimicking human tissue architecture and cellular responses, does not fully replicate the complexity of in vivo human colon tissue. Organoids lack several components of the immune system and the full diversity of cell types present in the human colon, which could affect the interpretation of our findings related to host–pathogen interactions and immune responses. Studies are needed to determine how accurately these in vitro models reflect the full range of GI symptoms observed in patients, including inflammation and other tissue-level responses associated with Mpox infections.

Second, our study focused on a limited number of samples per condition (three replicates for each Mpox strain and mock control). Although advanced statistical and machine learning techniques, like GLMQL-RMAS and Cross-RMAS, were employed to maximize the reliability of our findings, the small sample size inherently limited the statistical power of this study. Larger sample sizes would provide more robust validation of the identified differentially expressed genes and improve the generalizability of the machine learning models.

Additionally, the choice of viral strains, Clade I (Zr-599), Clade IIa (Liberia), and Clade IIb (2022 MPXV), could influence the observed phenotypic outcomes. The expression differences noted in our findings may be partially dependent on these specific strains, highlighting the intricate dynamics of viral–host interactions that could vary with different viral genotypes. Incorporating additional strains, such as those from Clade Ib, could provide a more comprehensive understanding of the spectrum of host responses, revealing further nuances in host–pathogen interactions and phenotypic manifestations.

Finally, while the GO analysis and machine learning models offer insights into potential therapeutic targets and diagnostic markers, the functional relevance of the identified genes requires further experimental validation. In vitro findings should be complemented with in vivo studies and clinical data to confirm the applicability of these markers for therapeutic or diagnostic purposes.

Addressing these limitations in future research will be essential for enhancing our understanding of Mpox pathogenesis and improving the translational relevance of findings derived from organoid models.

## Figures and Tables

**Table 1:** Comparative analysis of study findings and methodologies between our study and Watanabe et al. [13].

Category	Watanabe et al. [13]	Our Study
Virus Strains Used	Clade I (Zr-599), Clade IIa (Liberia), Clade IIb (2022 MPXV).	Clade I (Zr-599), Clade IIa (Liberia), Clade IIb (2022 MPXV).
Primary Focus	Examining viral growth efficiency and host responses in human keratinocytes and colon organoids.	Detailed analysis of DEGs in colon organoids infected with various MPXV strains to understand GI manifestations.
Cell Models Used	Human keratinocytes; iPSC-derived colon organoids.	Focused exclusively on iPSC-derived colon organoids.
Data Source	RNA-seq data.	RNA-seq data.
Statistical Analyses	Student’s *t*-test, ANOVA.	Generalized Linear Models with Quasi-Likelihood F-tests and Relaxed Magnitude–Altitude Scoring (GLMQL-RMAS) tailored for RNA-seq data analysis.
Machine Learning Analyses	No machine learning approaches were reported.	Introduced the novel Cross-RMAS system for identifying key genes such as *TFF1*, *GSTA5*, *HSPA6*, *FOS*, *MPIG6B*, *F2* and *HP*, which effectively distinguish among different MPXV clades and separate them from mock samples. Employed these genes as inputs for linear classifiers, LR and SVM with a linear kernel, achieving 100% classification accuracy in distinguishing each clade and mock, demonstrated through three-fold stratified cross-validation.
Significant Genes in Colon Organoids	Less detailed specific gene identification for colon organoids, focusing more on the comparison of infection efficiency and cellular responses between keratinocytes and organoids rather than highlighting specific gene changes within the organoids.	Extensive analysis has identified differentially expressed genes (DEGs) in colon organoids exposed to various MPXV strains using the Cross-RMAS method. Key genes include *TFF1*, *EGR1*, *FOS*, *HSPA6*, *GSTA5*, and *SERPINA3*. Figure 6 highlights unique and common genes across different MPXV clades. For MPXV I, unique genes such as *TFF1*, *CRYBB1*, *CRYAA*, *VCAM1*, *A2M*, and *CRYBA4* were prominent, while common genes with MPXV IIa included *FOS*, *NR4A1*, *DNAJA4*, *SLCO2A1*, and *DNAJB1*. MPXV I also shared some common DEGs with MPXV IIb, such as *GSTA5*, *FABP1*, *AHSG*, and *PLA2G2A*, which were not common with MPXV IIa. All clades shared common DEGs such as *HSPA6*, *EGR1*, *HASPA1A*, and *HSPA1B*.
Findings on Infection Efficiency	MPXV IIb shows productive infection mainly in keratinocytes, less so in colon organoids.	Clade I shows significant gene expression changes, with upregulation of stress-related genes such as *HSPA6*, *EGR1*, *FOS*, and *TFF1* and downregulation of genes crucial for cellular structure and signaling like *PSAP* and *CFL1*, indicating a disruption in cellular integrity and signaling that could affect infection outcomes. Conversely, Clades IIa and IIb exhibit fewer alterations, suggesting a more contained viral interaction with host cells. Clade IIa maintains a similar stress response to Clade I, while Clade IIb, showing minimal changes, may indicate either more efficient host defense evasion or less severe pathogenicity. The upregulation of defense genes like *SERPINA3* and *GSTA5* in Clade IIb points to specific protective responses against oxidative stress and protease activity.
Unique Observations	MPXV IIb-infected keratinocytes show increased expression of hypoxia-related genes.	Clade-specific gene expression profiling revealed critical pathways affected by each strain.
Pathway Insights	Hypoxia-related gene expression changes in MPXV IIb-infected keratinocytes.	Detailed GO analysis identified pathways like protein folding, immune response modulation, and cellular stress responses affected by MPXV.
Overall Findings	Demonstrated that MPXV replicates more effectively in keratinocytes than in colon organoids, with specific insights into the unique cellular responses of the MPXV IIb strain in keratinocytes.	Revealed distinct and detailed gene expression changes across MPXV strains in colon organoids using RNA-seq-tailored statistical and machine learning methods, highlighting the potential for strain-specific therapies.

## Data Availability

The RNA-seq data supporting the findings of this study, originally generated by Watanabe et al. [13] are openly available in the Gene Expression Omnibus (GEO) repository hosted by the National Center for Biotechnology Information (NCBI). These data can be accessed at https://www.ncbi.nlm.nih.gov/geo/ (accessed on 3 June 2024) under reference number GSE219036.

## Supplementary Materials

The following supporting information can be downloaded at: https://www.mdpi.com/article/10.3390/ijms252011142/s1.
