# Supplementary material for: Machine Learning Analysis of RNA-Seq Data Identifies Key Gene Signatures and Pathways in Mpox Virus-Induced Gastrointestinal Complications Using Colon Organoid Models
Source: Int J Mol Sci. 2024 Oct 17;25(20):11142. doi: 10.3390/ijms252011142 (PMC11508207; doi:10.3390/ijms252011142)
Supplement: Supplementary file 1 [file ijms-25-11142-s001.zip › ijms-3249130-supplementary.pdf]

Supplementary materials for

Article

Machine Learning Analysis of RNA-Seq Data Identifies Key Gene Signatures and Pathways in Mpox Virus-Induced Gastrointestinal Complications Using Colon Organoid Models

Mostafa Rezapour [1,*], Aarthi Narayanan [2] and Metin Nafi Gurcan [1]

[1] Center for Artificial Intelligence Research, Wake Forest University School of Medicine,Winston-Salem, NC 27101, USA; mgurcan@wakehealth.edu
[2] Department of Biology, George Mason University, Fairfax, VA 22030, USA; anaraya1@gmu.edu
* Correspondence: mrezapou@wakehealth.edu

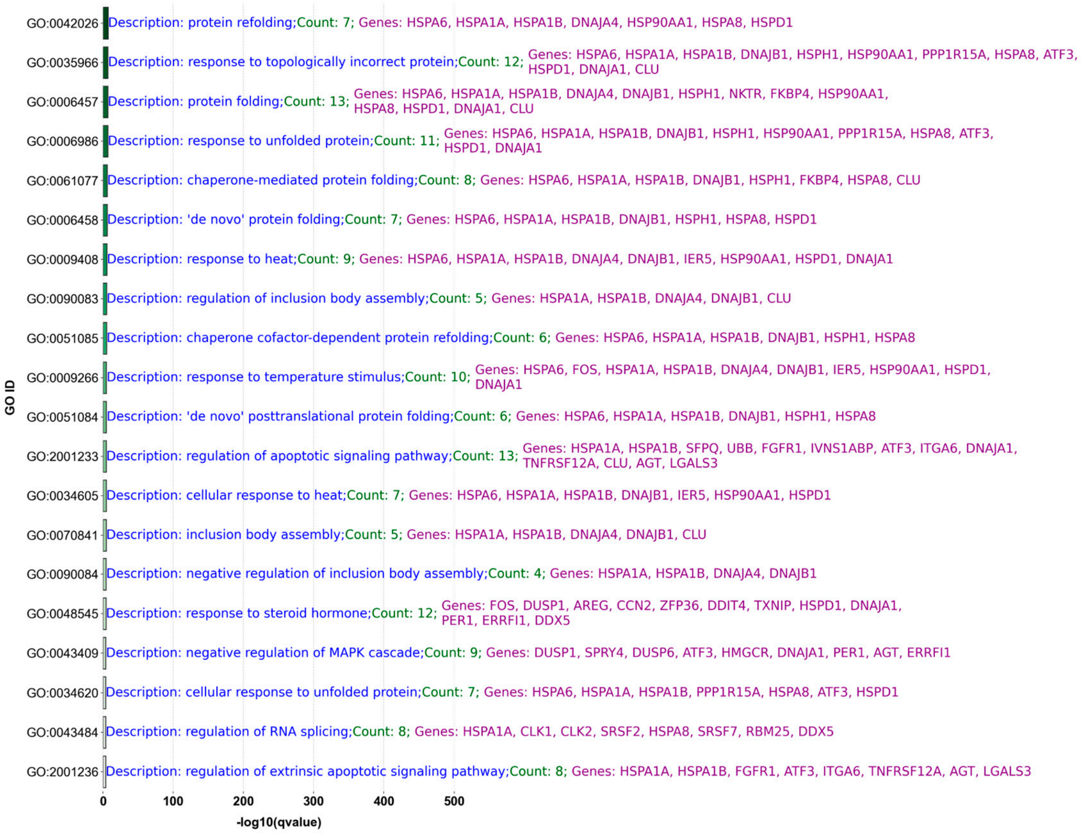

Figure S1: Top 20 GO biological processes linked to genes upregulated in MPXV IIa compared to Mock. The diagram details key biological activities affected by the MPXV IIa strain, offering a deeper understanding of its pathogenic mechanisms. This representation includes genes deemed significant before the application of the Benjamini-Hochberg adjustment, allowing for a broader inclusion of differentially expressed genes.

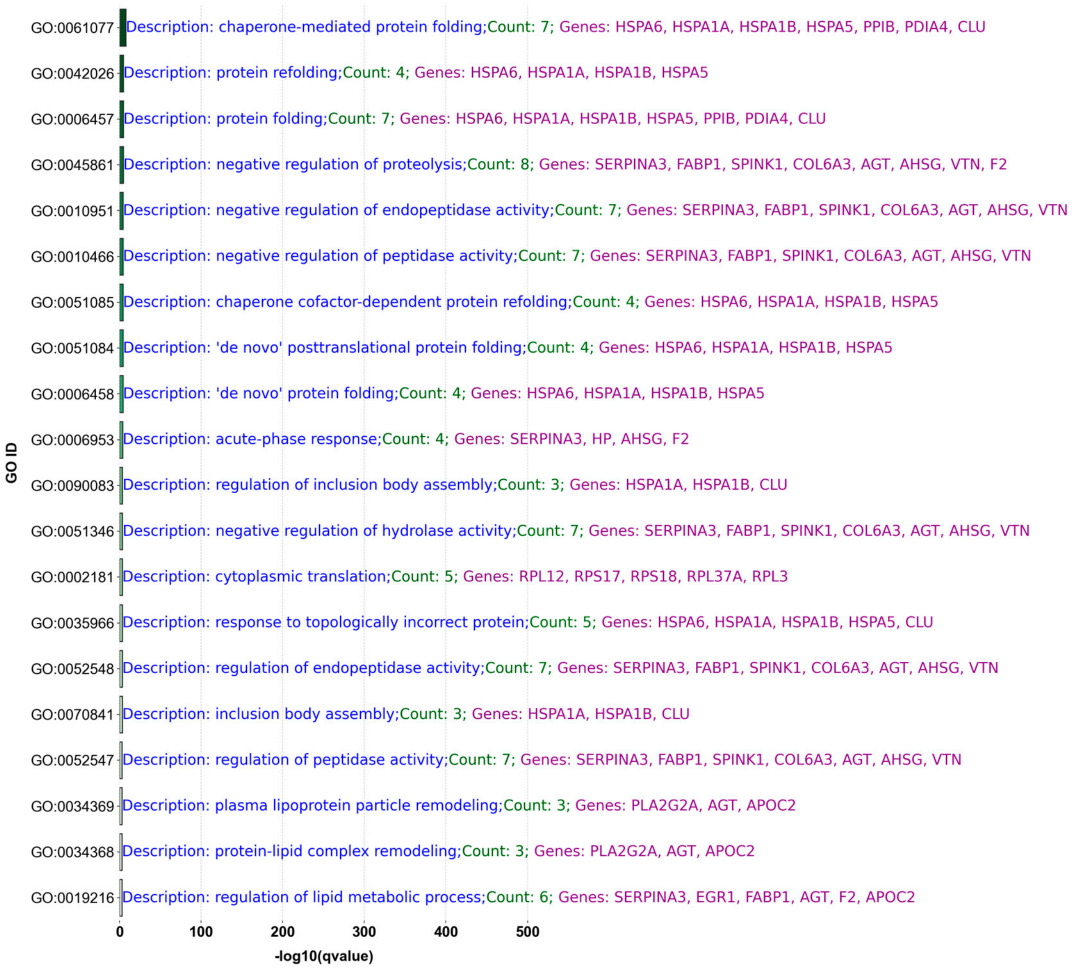

Figure S2: Top 20 GO biological processes associated with genes significantly upregulated in MPXV IIb compared to Mock. This figure captures the distinct biological pathways influenced by the MPXV IIb infection, highlighting potential targets for therapeutic intervention. This representation includes genes deemed significant before the application of the Benjamini-Hochberg adjustment, allowing for a broader inclusion of differentially expressed genes.
